# Bringing science to the public in the light of evolution

**DOI:** 10.1093/biomethods/bpad040

**Published:** 2023-12-18

**Authors:** Marie-Claude Blatter, Monique Zahn-Zabal, Samuel Moix, Béatrice Pichon, Christophe Dessimoz, Natasha Glover

**Affiliations:** SIB Swiss Institute of Bioinformatics, Lausanne and Geneva, Switzerland; SIB Swiss Institute of Bioinformatics, Lausanne and Geneva, Switzerland; SIB Swiss Institute of Bioinformatics, Lausanne and Geneva, Switzerland; Department of Computational Biology, University of Lausanne, Switzerland; SIB Swiss Institute of Bioinformatics, Lausanne and Geneva, Switzerland; Department of Computational Biology, University of Lausanne, Switzerland; SIB Swiss Institute of Bioinformatics, Lausanne and Geneva, Switzerland; Department of Computational Biology, University of Lausanne, Switzerland; SIB Swiss Institute of Bioinformatics, Lausanne and Geneva, Switzerland; Department of Computational Biology, University of Lausanne, Switzerland

**Keywords:** evolution, bioinformatics, science communication

## Abstract

Evolution stands as a foundational pillar within modern biology, shaping our understanding of life. Studies related to evolution, for example constructing phylogenetic trees, are often carried out using DNA or protein sequences. These data, readily accessible from public databases, represent a treasure trove of resources that can be harnessed to create engaging activities with the public. At the heart of our project lies a collection of “stories” about evolution, each rooted in genuine scientific publications that furnish both biological context and supporting evidence. These narratives serve as the focal point of our LightOfEvolution.org website. Each story is accompanied by a dedicated “Your Turn to Play” section. Within this section, we furnish user-friendly activities and step-by-step guidelines, equipping visitors with the means to replicate analyses showcased in the highlighted publications. For example, the website OhMyGenes.org, relying on authentic scientific data, provides the capability to compute the proportion of shared genes across different species. Here, visitors can address the captivating question: “How many genes do we share with a banana?” To extend the educational reach, we have developed a series of modular activities, also related to the stories. These activities have been thoughtfully designed to be adaptable for face-to-face workshops held in classrooms or presented during public events. We aim to create stories and activities that resonate with participants, offering a tangible and enjoyable experience. By providing opportunities that reflect real-world scientific practices, we seek to offer participants valuable insights into the current workings of scientists “in the light of evolution.”

## Introduction

Evolution is one of the cornerstones of modern biology. Charles Darwin played a key role in developing the theory of evolution, especially as it pertains to natural and sexual selection. He also presented a visual representation depicting the relationships between species and the concept of descent with modification, which constituted the only illustration in his landmark book “On the Origin of Species.” Such a tree, now called a phylogenetic tree, can be used to illustrate that present-day species have their origin in other preexisting species, with distinguishable differences being due to modifications throughout successive generations. In this way, the notion that all living creatures are related by descent from a common ancestor is depicted.

Historically, these studies relied on morphological, anatomical (i.e. fossils), and physiological characteristics. However, with advancements in DNA sequencing technology, the process of sequencing entire genomes is cheaper and faster. These genome sequences are easily and freely accessible from public sequence databases, bringing genomics—the study of the entirety of an organism’s genes—to the forefront of biological research. Genomics has revolutionized our understanding of evolution, including the construction of phylogenetic trees using DNA or protein sequences. Today, phylogenetic trees, boosted by genomic data, have become an essential tool of modern biological studies. They have diverse applications ranging from studying biodiversity and identifying new species to tracking viral outbreaks and their implications for vaccine development. Furthermore, they play a crucial role in forensic science, investigating human ancestry, and in the study of tumor cell evolution for precision medicine. The principles of phylogeny and genomics lie at the heart of many societal questions and health issues that have a direct impact on everyone.

In this publication, we present “In the Light Of Evolution” (LOE), a science communication initiative launched in May 2021 and spanning 3 years. The project’s primary objective is to engage individuals of all ages (starting from 9 years old) in the exploration of various aspects of evolution and the methods to study it, which involve the use of DNA, protein sequences, and bioinformatics.

Much research in education points to the importance of using a combination of multiple learning approaches including the “learning by doing” strategies [1, 2], and to “engage in inquiry activities similar to those of scientists” [3–6]. A challenge often encountered when dealing with subjects such as evolution is the complexity of accessing accurate and relevant datasets, primarily due to the vast amount of data available. Our LOE project aims to offer digested datasets related to scientific publications that are readily adaptable for educational purposes. We want to give people, including the youngest, a more concrete taste of the scientific process related to the study of evolution using molecular data and bioinformatics. We aim to offer them the chance to engage with genuine data for the purpose of exploring, testing, and validating hypotheses.

To accomplish this goal, we created two websites that host a series of easy-to-understand articles and interactive activities about evolution, genomics, and phylogenetics: In the Light of Evolution (https://lightofevolution.org/en/) and OhMyGenes! (https://ohmygenes.org/). These websites were used in the related modular workshops that were developed to present the activities face-to-face in the classrooms or during science fairs. This LOE project complements other existing initiatives such as the University of California, Berkeley’s “Understanding Evolution” [7], the Tree of Life Web Project [8], and the Swiss “The Grand Bazaar of Evolution” exhibition [9]. A key characteristic of LOE is our dedication to providing comprehensive, ready-to-use molecular datasets related to specific biological contexts, an approach that aids in understanding scientific methods in genomics and bioinformatics. Additionally, LOE aims to engage in a face-to-face dialog with the public through our interactive workshops.

## Approach to “In the Light of Evolution”

Demystifying something as complex as biology, evolution, and bioinformatics is not an easy task. In “In the Light of Evolution,” we employ a methodological approach, focusing on converting abstract concepts into tangible outcomes. To do so, our scientific communication project stands on two foundational pillars: first, defining and articulating scientific questions in a manner that can be grasped by non-specialists; second, applying real scientific methods, in other words, using real molecular data, methodologies, and literature and making them accessible and digestible.

**Figure 1.**
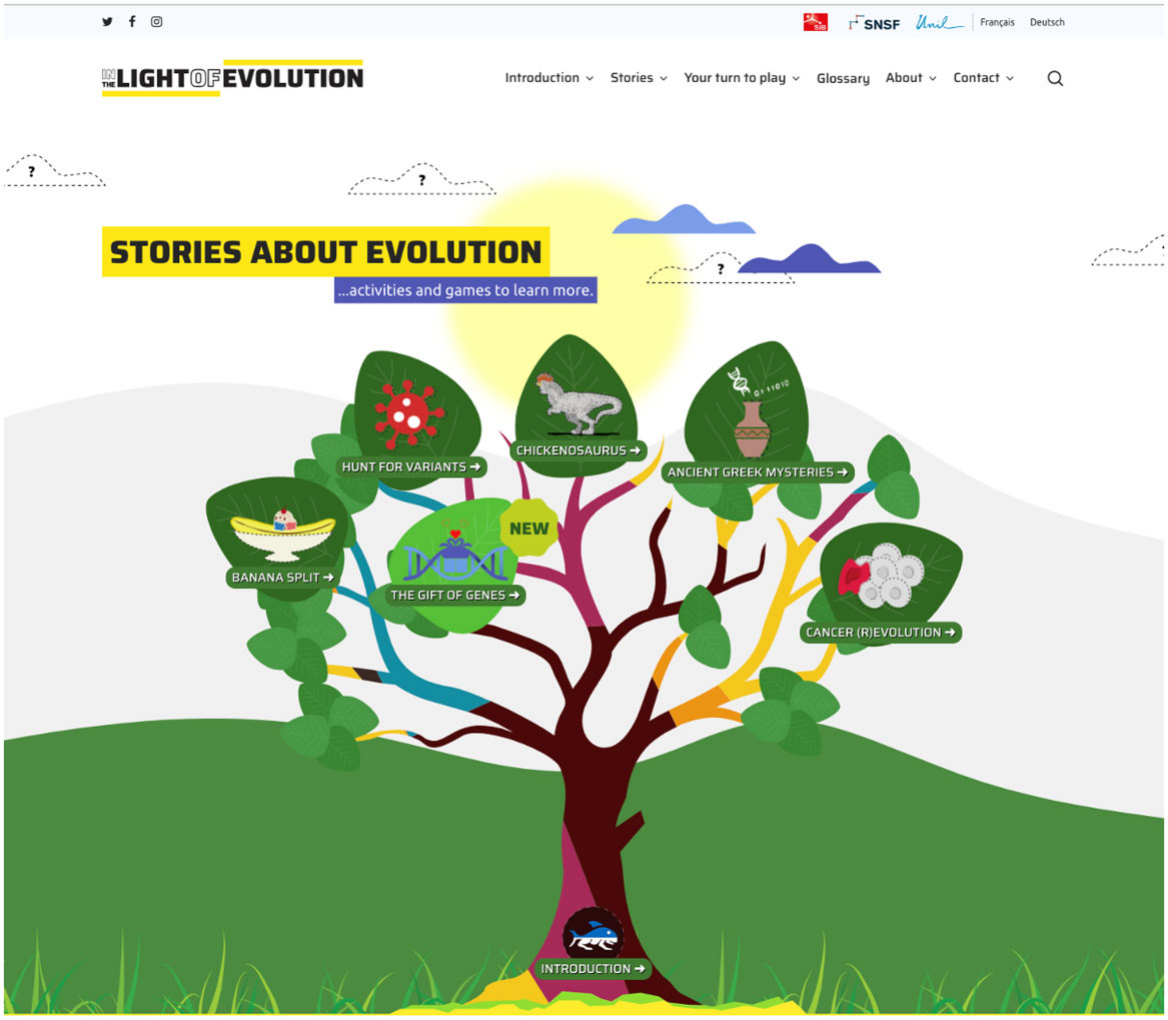
Screenshot of the “In the Light of Evolution” home page at https://lightofevolution.org/en/ (26 September 2023, date last accessed). Each leaf on the “Tree of Life” is a story, with the Introductory materials at the trunk, serving as a foundation. The main features of the website are accessible from the home page: the Introductory materials, Stories, Your turn to play (Activities), and the Glossary.

As mentioned above, the principal outcome of the project is the website https://lightofevolution.org/ (Fig. 1). This web portal serves to guide visitors through the discovery of different topics pertaining to evolution. Key features include:

- Introduction: Provides foundational knowledge on biology, evolution, and bioinformatics.
- Stories: Science articles on various topics linked to evolution in an easily digestible format, backed by scientific evidence.
- Your turn to play: Interactive, reusable, online, or pen-and-paper activities.
- Glossary: Definitions of key concepts fundamental to understanding the stories.
- Multilingual accessibility: Content translated into English, French, and German.
- Unified aesthetic: Professional graphic artistry.
- Open accessibility: CC-BY 4.0 license, ensuring free and reusable access to all.

### Stories: shining a light on evolutionary topics

The stories are at the core of In the Light of Evolution’s communication strategy and are added periodically throughout the project. All of our stories are based on actual scientific publications, which provide biological context and evidence. Each story is centered on a particular topic—focusing heavily on genomics and bioinformatics—but always linked to evolution.

Topics for our educational content were carefully chosen based on several criteria. We primarily selected peer-reviewed articles that utilized data from resources developed by the LOE team or our collaborators. In this way, both the original data and the analytical tools featured in our workshops are directly accessible through our websites (uniprot.org, omabrowser.org). Additionally, we focused on timely and relevant articles, such as those discussing the emergence of new coronavirus variants, to encourage current and engaging discussions. Last but not least, we also selected stories that are linked to a funny or thought-provoking biological question related to broader evolutionary concepts, such as “What is the link between T. Rex and chicken?”. As our philosophy is to create evidence-based materials, we use our own scientific expertise (genomics, phylogenetics, proteins, evolutionary biology, and genetics) and work directly with the authors of the publication, when feasible, or with partner scientists who also review our stories. Each story undergoes a thorough review by at least three scientists.

Our website, as well as the workshop material, is updated regularly according to the latest scientific news or participants’ feedback. Actively incorporating user feedback to create additional materials helps us to foster a collaborative learning environment, expanding our reach, and effectively catering to an even broader audience.

To date, we have published six stories, with several more planned. An example of the elements included in a typical Story is shown in Box 1.

Box 1. An example of a Story and its major elements: “The Gift of Genes”

This story can be found at https://lightofevolution.org/en/the-gift-of-genes/.

*Context and question(s)*

We start each story by providing the context and introduce the question that we wish to address. In the Gift of Genes story, we aimed to shed light on the concept of horizontal gene transfer (HGT) and its impact on the evolution of various species.

*Inspiration and background*

The story was inspired by the recent publication by [10] entitled “Discovery of archaeal fusexins homologous to eukaryotic HAP2/GCS1 gamete fusion proteins.” An introductory section provides essential concepts: defining HGT, elaborating on its importance, and introducing the provocative idea that horizontal gene transfer might have been instrumental in the emergence of sexual reproduction.

*Structural composition of the story*

The story itself is organized into concise sections that collectively elucidate the subject matter. These include discussions on:

- the origins of sexual reproduction;
- membrane fusion and fusogen proteins;
- identification of Archaeal fusexins and how researchers proved it;
- HGT and the evolutionary origins of sex; and
- did Archaea give us sex?

*Integration of multimedia elements*

We integrate various forms of media into the stories to create a curated blend of textual information, interactive elements, and multimedia to promote a dynamic and engaging learning experience.

Images

We work with a graphic designer to create original graphics or to find royalty-free stock images to accompany our stories. The website has its own distinct style, providing a cohesive and professional look. Shown below is an example of an original illustration.

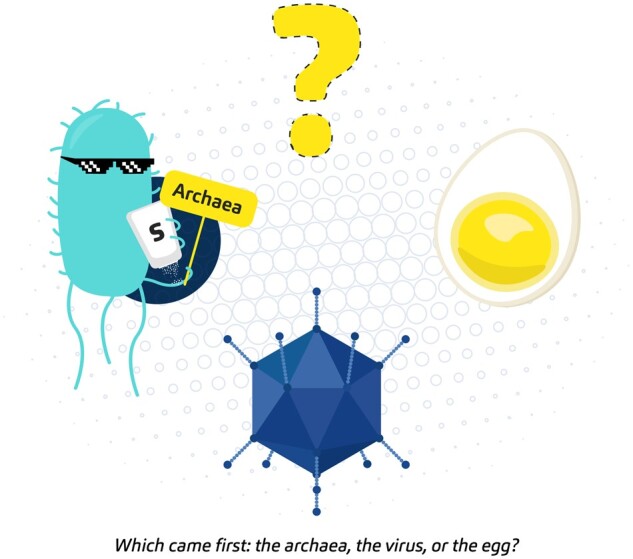

Interactive drop-downs

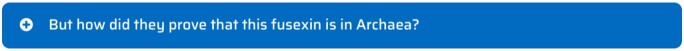

Some sections on the website are presented as interactive “click-to-display” components to leave it up to the reader to delve deeper into particular areas or not. These sections can be accessed by clicking the “+” button to see more. In this story, the “How did they prove that this fusexin is in Archaea?” section is in an interactive drop-down, as it is not essential to understand the essence of the story.

Videos

We incorporate relevant, short videos into our website. In The Gift of Genes, videos of gamete fusion, taken directly from the research paper [10], illustrate the fusion process in striking color.

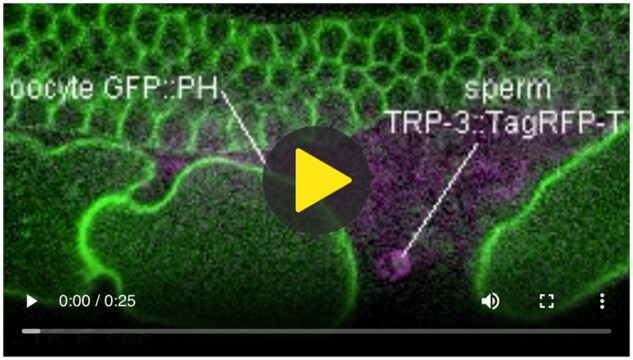

3D structures

The interactive 3D structure of the fusexin protein from the Protein Data Bank (PDB accession 7P4L) is embedded directly into the website for exploration of its structural conformation.

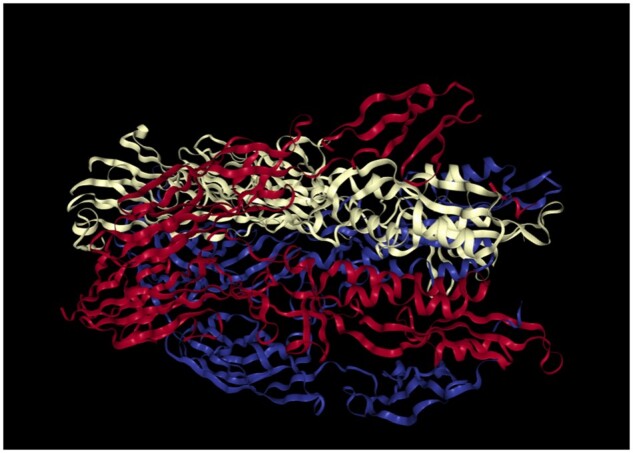

Animated GIFs

We also use animated gifs to inject liveliness and visual intrigue into the stories.

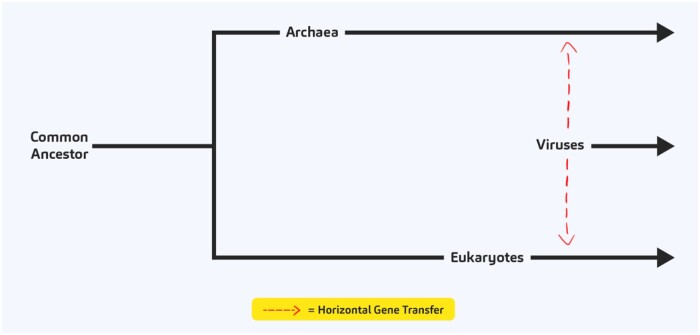

(In the image above, the red arrows are animated to show the directionality of the HGT.)

We offer a fact-based, comprehensive, and captivating insight into complex scientific topics, making them accessible and appealing to a broad audience.

### Your turn to play: activities making science tangible

Each story is accompanied by companion activities, which can be accessed by clicking on the “Your turn to play” link provided alongside the story. Our activities, which include both pen and paper as well as computer methods, recapitulate techniques used in authentic scientific research practice. Participants interact with actual DNA or protein sequences, using freely accessible bioinformatic tools or databases like OMA [11], UniProt [12], and BLAST [13]. As with the stories, all the activities are based on real data. For this, we curate specific examples that draw on publicly available data that may be hard to understand for the layperson.

We seek to simplify the methods as much as possible by reducing them to their essential elements. For example, instead of using whole protein sequences, we use short extracts of the sequence (∼20 amino acids). Whenever necessary, we guide participants step-by-step on how to use online professional resources. Furthermore, we provide comprehensive explanations of the resultant outcomes, ensuring a thorough understanding. As summed up eloquently, “Our main objective is to engage the layman in activities that are similar to authentic scientific research practice, and not to get lost in the technical know-how” [14].

An example of the elements included in a “Your turn to play” section is shown in Box 2.

Box 2. Example of “Your turn to play” for the “Banana split” story, using the OhMyGenes website

The goal of this activity is to discover the concepts related to comparative genomics and, more specifically, the quest for orthologs, to address the question: how many genes do we share with banana?

Each species has its own jar. The different genes for each species are represented by marbles.

The number of marbles is proportional to the number of genes in each species: each marble corresponds to about 1000 genes. The genes specific to each species are represented by the white marbles. The orthologous genes, which are common to the two species, are represented by colored marbles. The total number of genes and the total number of genes in common (orthologs) are proportional to the real data from OMA, an orthology database [11]. The aim is to calculate the percentage of orthologous genes between the two species.

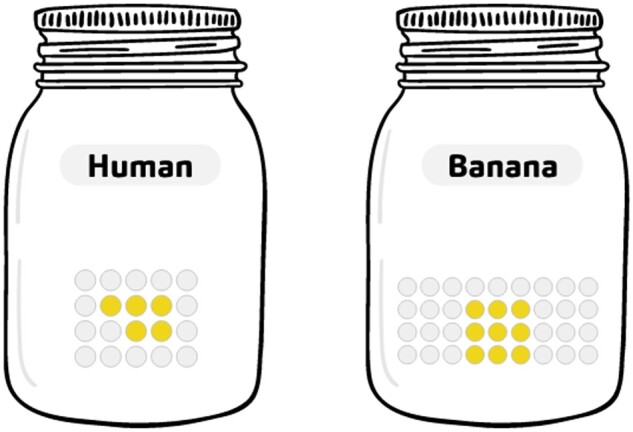

After gaining a clear understanding of the principles of the calculation with this quest for orthologs using marbles activity, participants proceed to verify their results on the OhMyGenes.org website.

OhMyGenes (https://ohmygenes.org/) is a website where users can find the percentage of genes shared by two species. One can choose the two species from a drop-down list of selected organisms and find out the percentage of genes in common between them. One can also explore the real scientific data by clicking the button to see how this number is computed: the mean of the percentage of genes in species A with an ortholog in species B, and the percentage of genes in species B with an ortholog in species A. These data are taken directly from OMA, using the API function “shared ancestry” (https://omabrowser.org/api/docs#summary-shared_ancestry-read). We also provide the estimated divergence time between two species, taken from Timetree.org [15]. Inspired by Internet memes, users can also create their own memes based on the percentage of shared genes.

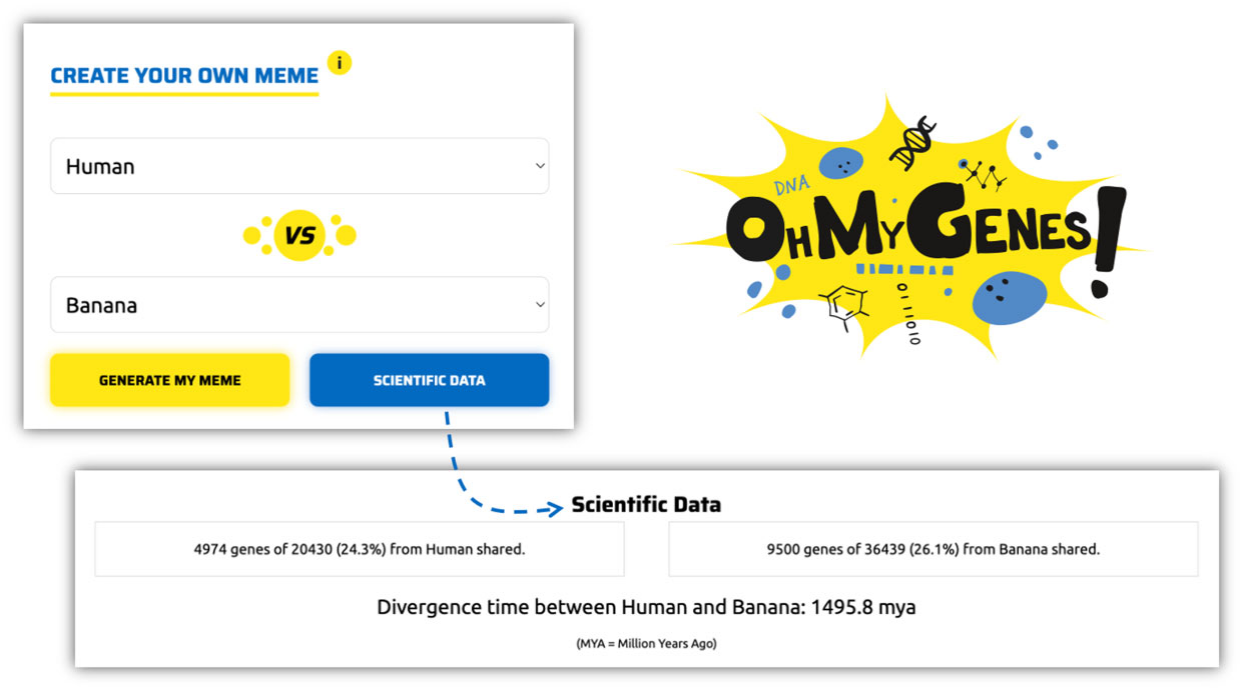

We share ~27% of our genes with banana.

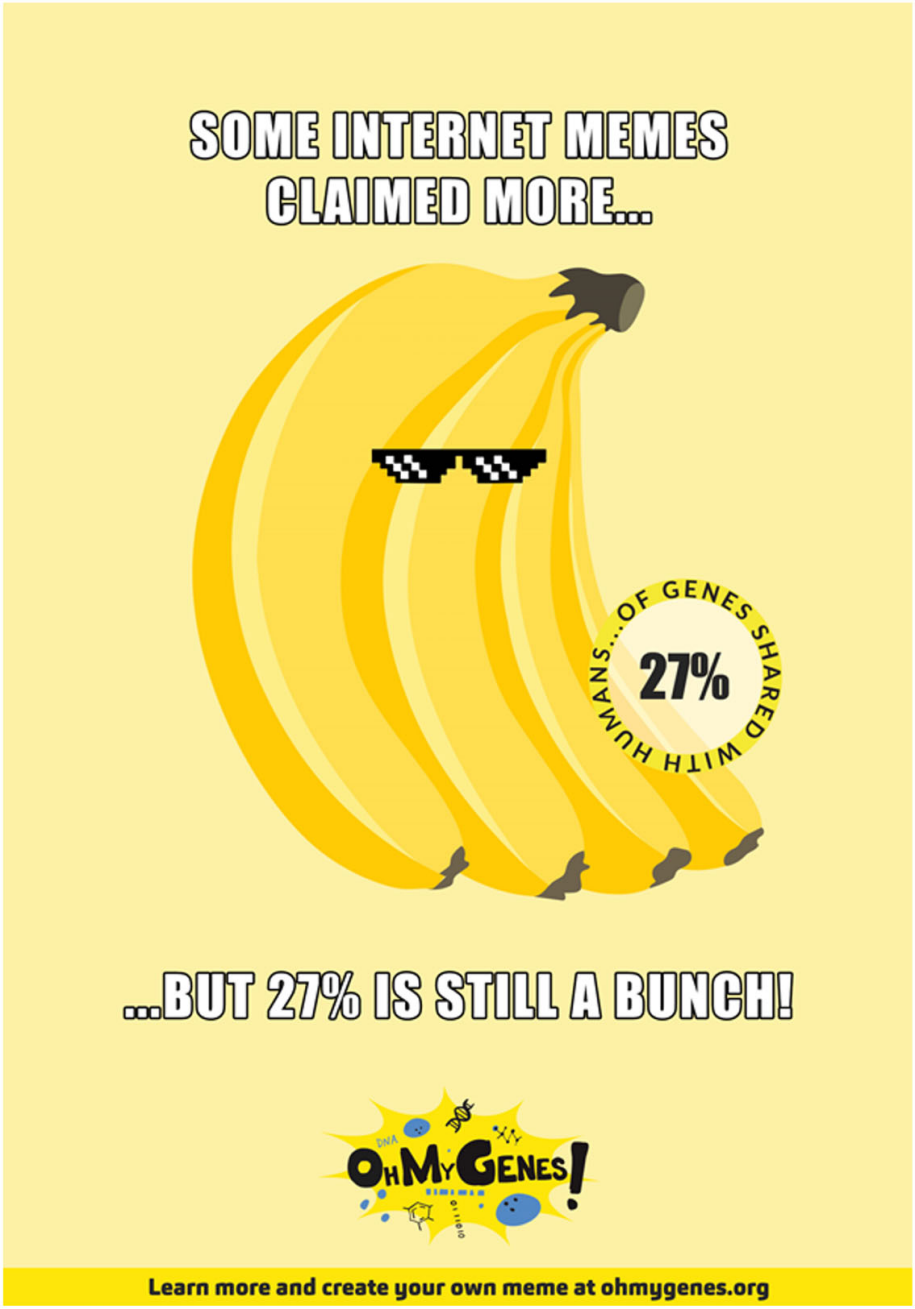

Each story, associated “Your turn to play” activities, its lede, learning objectives, and associated databases & tools are listed in Table 1.

**Table 1.** Stories, lead, learning objectives, and databases & tools used.

Story	Lede	Learning objectives		Databases and tools
		Story	Your turn to play	
Hunt for Variants	Alpha, Beta, Gamma, Delta, Omicron… what’s that? Discover SARS-CoV-2, its variants, and their impact on the pandemic	- Describe what is a virus - Visualize the SARS-CoV-2 genome - Explain what a mutation is and what is meant by “SARS-CoV-2 variants” - Explain what is the SARS-CoV-2 spike protein and its 3D structure - Explain the impact of the spike N501Y mutation - Explain how the evolution of the virus can be deduced from mutations - Describe the surveillance of SARS-CoV-2 variants	- Find differences between SARS-CoV-2 genomes - Use the genetic code to find the mutations in the SARS-CoV-2 spike protein - Track the evolution of the virus from the SARS-CoV-2 variant sequences - Determine if the spike N501Y mutation is present in a given SARS-CoV-2 variant found in wastewater	GenBank PDB Nextstrain UniProt ViralZone
Banana Split	Did you know we share about 98% of our genes with chimpanzees? But how many with the banana?	- Recognize that species may have a common ancestor - Describe what orthologs are - Explain how orthologs are detected - Recognize that different methods can give different % of genetic material in common between species - Discover the biological functions of orthologous proteins	- Determine the % of genes in common between different species - Discover the OhMyGenes.org web site	OMA UniProt
Chickenosaurus	Dinosaurs haven’t all disappeared? Really?	- List key dates in the history of life on earth - Discover proteins from species which are extinct today - Explain how ancient proteins can be used to study the relationship between different species - Describe the classification of dinosaurs and birds based on the sequence of their collagen proteins	- Compare the sequences of the collagen protein from different species (use substitution matrix) - Place species on a phylogenetic tree based on the differences in protein sequences	Align SIM UniProt
Ancient Greek Mysteries	What does their DNA tell us?	- List key dates in ancient Greek history - Explain how ancient DNA can be used to track the origins of human populations - Recognize that every human being has a unique genome - Explain how Multidimensional Scaling (MDS) is used to visually represent the similarity between individuals - Find the genetic relatedness among Aegean Bronze Age civilizations	− Determine what the ancient Greek individuals living in the Bronze Age looked like, using popular SNPs	GenBank OMIM UniProt
Cancer (R)evolution	Fighting cancer means fighting evolution… a (r)evolution?	- Explain what is a cancer cell - Explain how cancer is a story of mutations - Explain how cancer cells evolve - Recognize different models to represent the evolution of cancer cells - Recognize therapies against cancer inspired by the principles of evolution	- Match the types of evolution with the representations of the evolving cell populations within a tumor - Recognize that the same process takes place in bacteria exposed to antibiotics	UniProt
The Gift of Genes	Did Archaea give us sex?	- Describe what is horizontal gene transfer - Explain the role of membrane fusion in sexual reproduction - Describe what are fusogen proteins - State the evidence that fusexins also exist in prokaryotes - Describe the hypotheses for when did the genes coding for fusexins proteins arise and what is their evolutionary history	- Discover the origins behind the capacity of bacteria found in the Japanese gut to effectively digest sushi - Discover the origins of the protein syncytin and why without viruses, there might not have been any mammals on earth - Discover one mechanism by which insects develop resistance to plant defenses	BLAST PDB UniProt

## Workshops

We have designed a range of modular activities based on the different “Stories” and the “Your turn to play” sections, to use them in classrooms or during various public events. The modular structure enables easy adaptation to diverse audiences. It offers flexibility in terms of duration (ranging from 20 to 90 min), content, level of difficulty, learning styles, and alignment with different school curricula. At the end of the workshop, we always allow for time to foster dialog, discussions, and debates with the participants, as well as for participants to explore the two websites.

Our workshops are typically facilitated by one or more members of our team. The presenters are always people who work in a related field and who have been previously trained to present the activities. Depending on the person and their field of expertise, the topics highlighted may differ. This is essential, as it demonstrates to participants that even people in corresponding fields are unable to answer all questions. It conveys the message that we should recognize the limits of expertise and avoid trying to believe that it is possible to be competent in every field.

At the beginning of the workshop, there is a concise introduction to fundamental concepts related to DNA, genes, and proteins, and their relevance to studying evolution.

**Figure 2.**
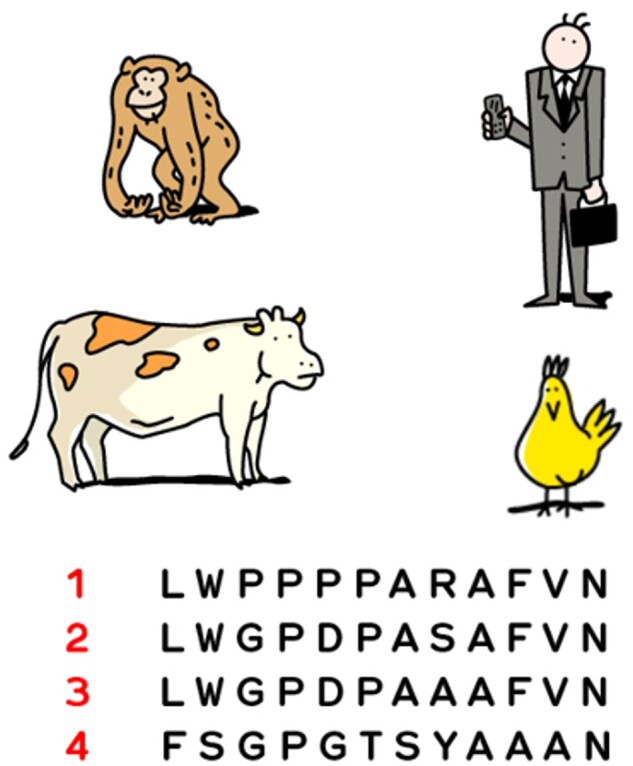
Partial multiple sequence alignment of insulin from different species (UniProt accessions P01308 (human), P30410 (chimpanzee), P67970 (chicken), P01317 (cow). In order to respond to the question “Which sequence belongs to which species?”, workshop participants must deduce which protein sequences are the most similar, and relate it to which species are morphologically the most similar. The human and chimpanzee sequences (2 and 3) are the most similar, with one difference. The chicken sequence (4) contains the most differences from the other sequences. By process of elimination, sequence (1) belongs to the cow.

Figure 2 presents an illustration highlighting the key concept of aligning protein sequences for studying evolution—the more similar the sequence, the more recent the common ancestor, and the more related the species. The related question “Which protein sequence belongs to which species?” serves as an excellent starting point to initiate a discussion on the “how to” approach.

As an example, we present three different workshop modules, which can be combined if desired. Box 3 depicts an example module titled “Finding the Orthologs,” a key step in phylogeny. During the introduction part, we explain that in Darwin’s time, species were first compared based on their morphology, that is the “same” skull bones of different species were compared. Today, it is possible to study the evolution of species by comparing their proteins. For this, it is essential to compare what is comparable—the same protein in different species, in other words, orthologous proteins.

Box 3. Example of a workshop module and its main elements: “Finding the Orthologs”

In this interactive exercise, participants engage in an activity known as “Finding the Orthologs.” The underlying data including protein sequences, orthologous groups, and protein functions are all taken from OMA, a database for orthologous groups amongst over 2500 species [11], and UniProt, a knowledgebase for over 248 million proteins [12]. This is how the workshop unfolds:

1. **Distribution of Materials**: Participants are given 20 strips of paper, each corresponding to extracts of protein sequences, which are derived from five species. A cartoon image identifies the corresponding species on each strip.
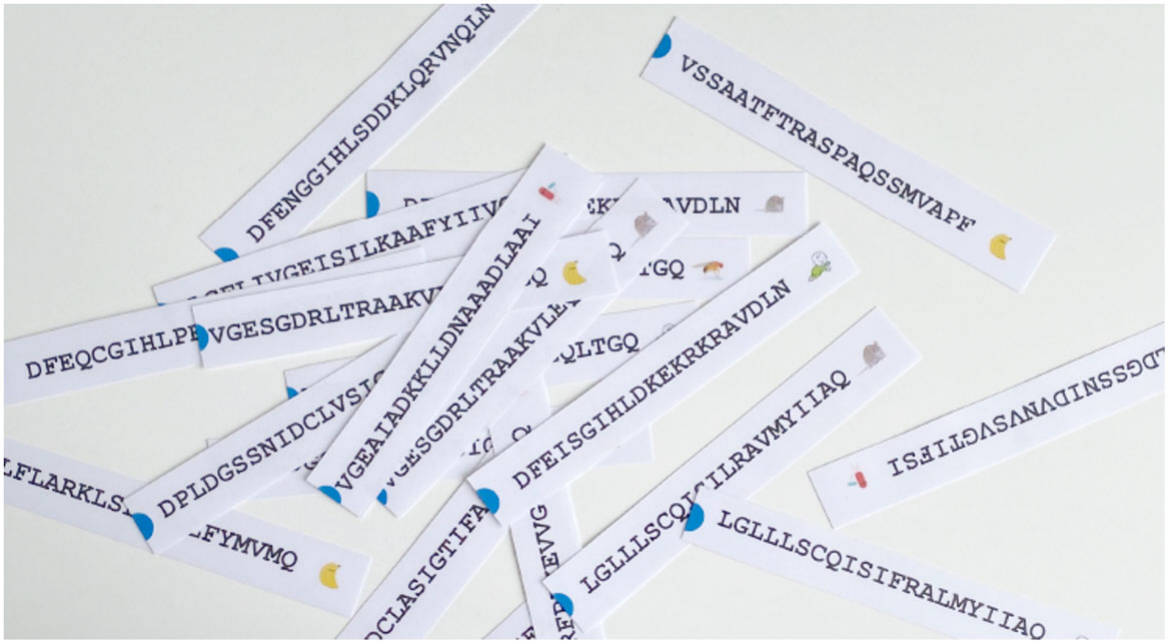
2. **Alignment Task**: Participants must classify these sequences into orthologous groups by creating “alignments.” However, one orthologous group lacks matching counterparts—a deliberate twist in the exercise.
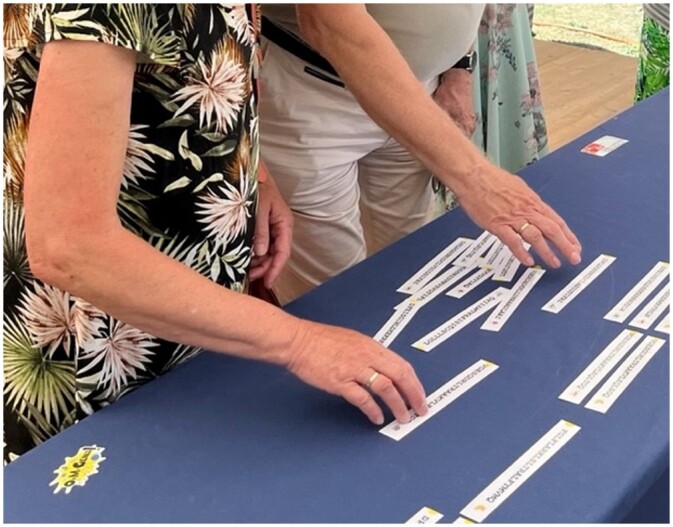
3. **Critical Observation**: This anomaly prompts the presenter to engage the participants in discussion, probing their insights. The participants often identify that the unmatched sequence belongs to a banana, leading some to infer that it's a species-specific sequence.
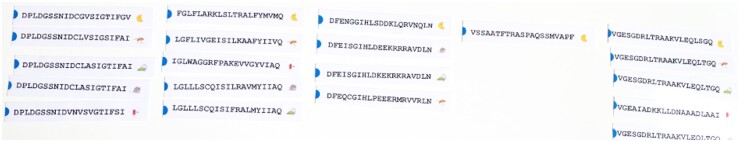
4. Using a miniature **BLAST**: The next phase involves conducting a “BLAST” against a small-scale UniProt “database” to uncover the function of the lone protein without an orthologous group. This database, crafted specifically for the activity, comprises 30 proteins printed on individual sheets, accompanied by illustrative visuals.
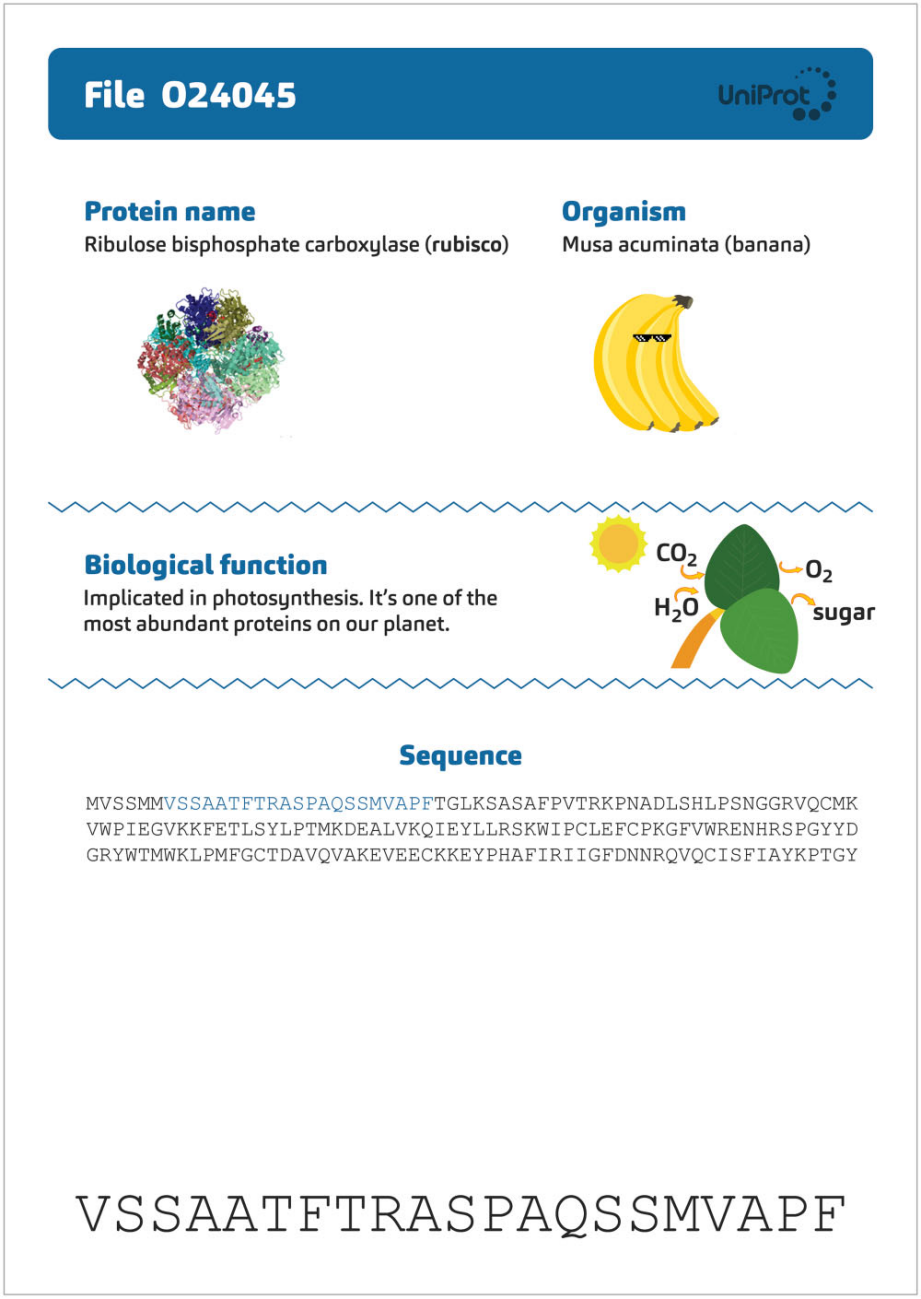
5. **Protein Identification Process**: Participants circulate among the sheets, matching their unique sequence to the correct paper, and thus identifying the specific protein.
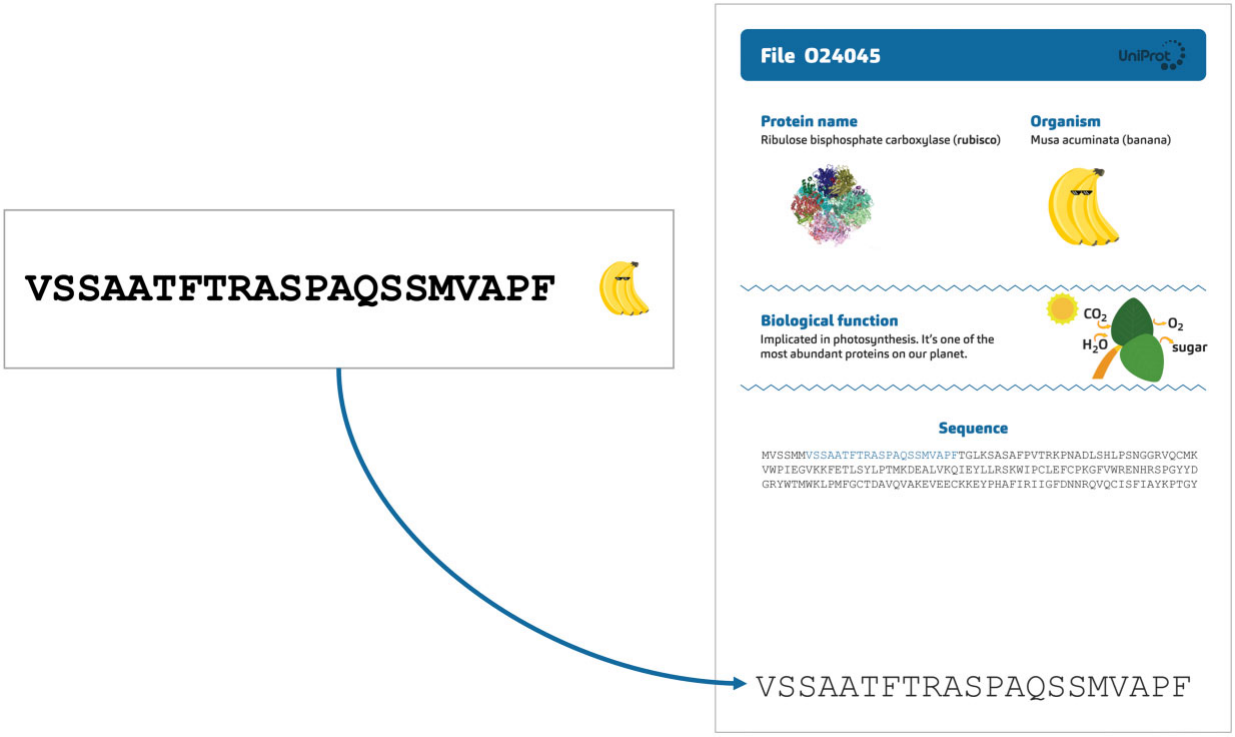
6. Revelation and Learning Moments: The participants discover the protein is rubisco (UniProt accession O24045), vital to photosynthesis, and mainly found in plants. This revelation serves as a springboard for instructive dialogues on key concepts:
  - How does one make an alignment?
  - Why might some orthologous groups exclude certain species?
  - What are the biological functions of these different proteins?
  - Which biological functions are shared across various groups of species (universal proteins, proteins specific to mammals, to insects, …)?
  - Why is bioinformatics important?

By combining hands-on activities with guided discovery and critical thinking, “Finding the Orthologs” offers an experiential learning environment. Participants are not merely told the scientific principles: they engage with them actively, making the abstract concepts of orthology, alignment, and protein function tangible and memorable.

**Figure 3.**
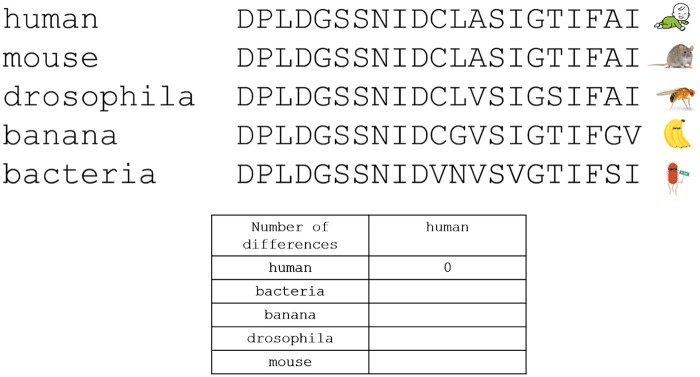
Partial multiple alignment of fructose-1,6-biphosphatase from different species (UniProt accessions: human O00757, mouse P70695, Drosophila A0A0U1RVK6, banana Q9XF47, and bacteria (in this case *Escherichia coli*) P0A993).

A second workshop module called “Look for the Differences” empowers participants to explore the distinct orthologous groups identified in the previous module and gain further insights from their analysis. Participants are provided with a sheet containing the multiple sequence alignments and a table to complete (Fig. 3). Each protein sequence is compared to the human sequence, and then participants proceed to fill in the corresponding table with the number of differences.

Based on their results, participants then determine which species is the most distinct from humans, identify the subsequent closest species, and so on. The discussions focus on validating these results which are derived from a limited number of short protein sequences, as well as considering any potential biases when using humans as the reference sequence.

In a third workshop module, “Construct a tree,” participants can build multiple protein sequence alignments starting from complete protein sequence sets and using online tools. They look at the corresponding trees to answer questions related to “who's whose cousin?”

For this module, we provide different sets of orthologous protein sequences. The protein sequences are obtained from UniProt, featuring a simplified FASTA header to avoid overwhelming technical details. These sequences can be directly copied/pasted to build a multiple sequence alignment using the UniProt Align tool. Based on the corresponding tree, participants can answer different questions. The collagen (gene COL1A1) sequence set can be used to determine how dinosaurs and birds are related (see the Chickenosaurus story). The question “From which species might SARS-CoV-2 have originated?” can be investigated by aligning the coronavirus spike protein sequence set (related to the Hunt for Variants story). The set of cytochrome B (gene MT-CYB) sequences allows the participants to answer fun questions such as “Who is the dodo’s cousin?” or “Who is the mammoth's cousin?” Alternatively, advanced participants can also perform direct queries on UniProt using a selection of gene names (gene: INS, gene: COL1A1, gene: TP53,…) and choose the species they wish to include in their multiple alignment.

Discussions during this module emphasize the importance of beginning with a clean set of orthologous protein sequences (of similar sequence lengths). The concept of “Finding the orthologs” can be further highlighted by using the first module described in Box 3. Participants will also be informed that the trees do not always correspond to the expected species trees due to the limited number of sequences/species analyzed and other evolutionary phenomena such as gene duplication, loss, and HGT. Additionally, the significance of utilizing robust statistical methods in tree construction is emphasized, along with the encouragement to always seek expert help in the field of phylogeny when required.

The materials and instructions for these different modules, “Find the orthologs,” “Look for the differences,” and “Construct a tree” are available at: https://education.expasy.org/cours/Outreach/LOE. Links are also available under the “Introduction” → “For the teacher” section of the LOE website and from the story “Banana Split.”

## Holding workshops at schools

The workshops are conducted regularly in school classrooms, with more than 20 sessions held each year. Through pilot sessions conducted first with selected classes, we can effectively tailor the content of our workshops to meet the specific needs and interests of teachers and students alike. One of our primary objectives is to ignite and channel scientific curiosity among students. By providing engaging experiences, we aim to help students explore and uncover new areas of research, ultimately creating a passion for scientific inquiry.

In this digital age, high-school teachers are actively seeking innovative teaching and learning strategies, particularly in the STEM (science, technology, engineering, and mathematics) fields. We provide regular high-school teacher training for both public and private schools to empower educators and encourage sustainability in our initiatives. Equipped with the necessary skills, teachers can confidently conduct these activities with their classes independently, in a recurring fashion, and even have the potential to further enrich and expand upon them.

We aim to present exciting examples that highlight the importance of multidisciplinary approaches in tackling biological problems, standing out from traditional classroom activities.

## Holding workshops for the public

Our workshop modules are regularly showcased during science fairs, university open house days, or global events (such as Pint of Science), which allows us to directly engage with the public. Feedback from participants has been overwhelmingly positive, with many expressing their appreciation for the concrete, realistic, fun, and authentic nature of our activities. By engaging in hands-on bioinformatics exercises and using professional tools, participants were able to experience a genuine scientific process firsthand and to have valuable insights into the current workings of scientists.

## Spreading the word

Different approaches, venues, and communication channels were used to engage the broadest possible audience. As the SIB Swiss Institute of Bioinformatics has been active in outreach for 20 years, we have an extensive network that is actively involved in science outreach in Switzerland and beyond. Our workshops were advertised and/or organized through the Service Culture Mediation Scientifique (SCMS)/L’Eprouvette at the University of Lausanne (UNIL), Scienscope at the University of Geneva, Expanding Your Horizons, and the TecDays of the Swiss Academy of Engineering Sciences (SATW). Science fairs in Lausanne and Geneva proved to be excellent venues to engage the general public during our workshops, as was the participation in the global Pint of Science initiative in 2023.

In terms of communication, each new story, presence at a science fair, or scientific congress was communicated using social media. To do so, we used X (https://twitter.com/LightOfEvol), Facebook (https://facebook.com/SIBbioinformatics/), Instagram (https://instagram.com/lightofevolution), as well as our personal LinkedIn accounts. We benefited from links from other science communication websites, such as Simply Science, or from online articles, such as the one in Snopes. The SIB communication team gave us additional visibility by posting several news items on the project on the institutional website; they also provided promotional materials (stickers, bookmarks, etc.). The project was also presented at several scientific conferences, including the Quest for Orthologs meeting in 2022, the Life Sciences LS2 Switzerland meeting in 2023, as well as the annual meeting of the Society for Molecular Biology and Evolution in 2023.

## Final thoughts

The “In the Light of Evolution” project captures the essence of bioinformatics in an engaging manner, illustrating that science can be both intellectually stimulating and fun. Our core strategy involves simplifying complex and relevant scientific concepts using accessible methods. Simultaneously, we recognize that fostering critical thinking skills is crucial to counteract the spread of scientific misinformation. So far, our project has successfully reached over 2750 participants across 85 events, highlighting its effectiveness. We are committed to highlighting a broad range of future career opportunities in science to young individuals, irrespective of gender.

To ensure the long-term sustainability of the LOE website, we have designed the “Your Turn to Play” activities to be self-explanatory, allowing them to be conducted without the need for an expert’s involvement. Although this autonomous model is not applicable for all our workshops, we have taken steps to ensure their continued availability and relevance. For example, the material and instructions for the modules are available on the LOE website, and we provide regular high-school teacher training to empower and encourage educators to reuse our materials. Additionally, we work with a number of motivated PhD students to manage ongoing workshops as part of their teaching duties, allowing them to gain important skills in scientific communication. Finally, we aim to maintain our partnerships with well-established, long-running initiatives such as Scienscope at the University of Geneva, where we have launched a workshop and conducted training for its mediators. We hope that these measures encourage sustainability in our initiatives.

As we conclude, the words of Theodosius Dobzhansky resonate: “Nothing in biology makes sense except in the light of evolution.” This principle reflects our project’s fundamental philosophy—that evolution is the unifying thread throughout biology.

## Data Availability

The data underlying this article are available at https://lightofevolution.org/, https://ohmygenes.org/, and https://education.expasy.org/cours/Outreach/Publication_SMBE/.
